# Interaction interface in the C-terminal parts of centriole proteins Sas6 and Ana2

**DOI:** 10.1098/rsob.200221

**Published:** 2020-11-11

**Authors:** Agnieszka Fatalska, Nikola S. Dzhindzhev, Michal Dadlez, David M. Glover

**Affiliations:** 1 Department of Genetics, University of Cambridge, Cambridge CB2 3EH, UK; 2 Division of Biology and Biological Engineering, California Institute of Technology, Pasadena, CA 91125, USA; 3 Institute of Biochemistry and Biophysics, Polish Academy of Sciences, 02-106 Warsaw, Poland

**Keywords:** Sas6, Ana2, centrosome, hydrogen–deuterium exchange mass spectrometry, centrioles, protein interaction

## Abstract

The centriole is a ninefold symmetrical structure found at the core of centrosomes and, as a basal body, at the base of cilia, whose conserved duplication is regulated by Plk4 kinase. Plk4 phosphorylates a single serine residue at the N-terminus of Ana2 to promote Ana2's loading to the site of procentriole formation. Four conserved serines in Ana2's STAN motif are then phosphorylated by Plk4, enabling Sas6 recruitment. Crystallographic data indicate that the coiled–coil domain of Ana2 forms a tetramer but the structure of full-length Ana2 has not been solved. Here, we have employed hydrogen–deuterium exchange coupled with mass spectrometry (HDX-MS) to uncover the conformational dynamics of Ana2, revealing the high flexibility of this protein with one rigid region. To determine the elusive nature of the interaction surfaces between Ana2 and Sas6, we have confirmed complex formation between the phosphomimetic form of Ana2 (Ana2-4D) and Sas6 *in vitro* and *in vivo*. Analysis of this complex by HDX-MS identifies short critical regions required for this interaction, which lie in the C-terminal parts of both proteins. Mutational studies confirmed the relevance of these regions for the Ana2–Sas6 interaction. The Sas6 site required for Ana2 binding is distinct from the site required for Sas6 to bind Gorab and Sas6 is able to bind both these protein partners simultaneously.

## 1. Introduction

Centrioles are ninefold symmetrical structures at the core of centrosomes and in the form of basal bodies, at the base of cilia, which when dysfunctional lead to a wide range of inherited diseases including ciliopathies and microcephaly, and which frequently show abnormalities in structure and number in cancer [1]. Centriole duplication is regulated by the conserved protein kinase, Polo-like kinase 4 (Plk4), whose levels and activity are critical to ensure cells have a single centrosome, each comprising a parent centriole and extended procentriole at each of their spindle poles during mitosis [2,3]. Depletion or inhibition of Plk4 leads to the loss of centrioles [4,5], and its overexpression can result in their de novo formation and overduplication [6–8]. Plk4 displays distinct modes of recruitment to centrioles in different model systems. In *Caenorhabditis elegans*, Zyg1 (the Plk4 homologue) is targeted to centrioles via its interaction with Spd2. In *Drosophila*, it is recruited via an interaction with another centriolar protein—Asterless (Asl). In mammalian systems, however, the concerted action of both of these proteins' homologues (Cep192 and Cep152, respectively) is required for the correct centriolar targeting of Plk4 [9–13].

Plk4 is initially recruited by Asl to multiple sites in a ring-like formation at the periphery of the centriole mirroring the ring-like distribution of Asl itself. Plk4 first phosphorylates a single serine residue, in a conserved region of Ana2 (*Drosophila*)/STIL(human) at its N-terminal part, which promotes Ana2 recruitment to the site of procentriole formation. In *Drosophila,* this phosphorylation takes place in telophase, before Plk4 finally becomes restricted to a single site. In a second step, Plk4 phosphorylates four conserved residues in the STAN motif in the C-terminal part of Ana2 leading to the recruitment of Sas6 by Ana2 [14–17]. Dimers of Sas6 assemble into ninefold symmetrical structures that form the basis for the cartwheel structure at the core of the procentriole [18,19]. Sas6 then interacts with Cep135/Bld10 and with Sas4 (*Drosophila*)/CPAP (human) providing a link to the centriolar microtubule wall [20–26]. We recently found that the C-terminal part of Sas6 binds Gorab, a trans-Golgi-associated protein, whose human counterpart is mutated in the wrinkled skin disease, gerodermia osteodysplastica [27–30]. Ana2 possesses a Sas4-binding site at its N-terminus, a coiled-coil (C–C) domain in the central part, and a STAN motif at its C-terminus. Ana2 forms a tetramer through its C–C region and interacts with Sas6 via the STAN motif [31–37]. Although a crystal structure of Ana2's C–C domain has been determined, the crystal structure of the full-length Ana2 has not been resolved. As both Ana2 and Gorab interact with the C-terminal part of Sas6, it becomes important to understand the relationship of these binding sites with each other. To gain more understanding about the structure of Ana2 and its interaction with Sas6, we have employed hydrogen–deuterium exchange monitored by mass spectrometry (HDX-MS) to obtain structural information of exposed regions of these molecules alone and in the complex. HDX enables monitoring of the exchange of main chain amide protons to deuterium in solution. In an HDX-MS experiment, the protein sample is incubated in the deuterium buffer for a given time. Main chain amide protons exchange with deuterium from the buffer at different rates depending, among other factors, on the dynamics of the hydrogen bonds in which they are involved. Regions of highly dynamic structure undergo rapid exchange, whereas rigid regions, with amides engaged in stable hydrogen bonding, exchange slowly. The level of exchange reveals the extent of dynamic structural elements within a protein. Moreover, protein–protein interactions can also affect exchange in nearby regions, close to interaction surfaces and allosteric changes can also be observed [38–42]. Here, we have reconstructed the Ana2–Sas6 complex *in vitro* and purified it using size exclusion chromatography. We have then used a combination of HDX-MS together with *in vitro* and *in vivo* binding assays and mutational studies to map the interacting surfaces within the Ana2–Sas6 complex. This confirms the interaction of Ana2's STAN motif with Sas6 and defines the region within Sas6's C–C responsible for that interaction. It also reveals that Sas6 can accommodate the binding of both Ana2 and Gorab through interactions at different sites.

## 2. Results and discussion

**Figure 1.**
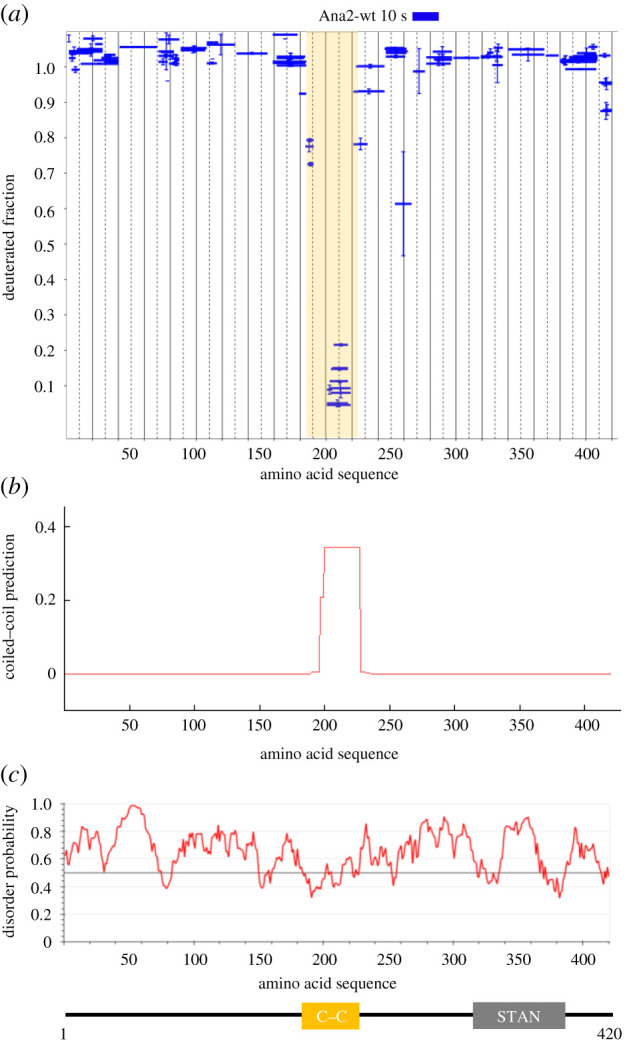
Ana2 is highly flexible, behaving as an intrinsically disordered protein. (*a*) Pattern of HDX in Ana2-wt peptides following a 10 s incubation with deuterium oxide (heavy water). Blue bars on Woods plots represent proteolytic peptides identified by mass spectrometry (MS) and positioned at the *x*-axis in relation to the Ana2 amino acid sequence, while their corresponding values of the fraction of deuteration (mean of two experiments) are shown at the *y*-axis. Error bars show both values measured. (*b*) C–C prediction generated by COILS software [43]. The *y*-axis shows the score of C–C probability; the *x*-axis shows the amino acid sequence. The C–C region aa195–229 matches the structured region identified by HDX. (*c*) Upper panel: prediction of intrinsically unstructured protein generated by IUPred2A software [44]. The *y*-axis shows the probability score of disorder; the *x*-axis shows the amino acid sequence. The program predicts the disordered character of Ana2 with high probability. Lower panel: representation of Ana2 showing known domains: yellow box, structured region identified by HDX-MS covering C–C motif; grey box, STAN motif.

Knowledge of the organization of the Ana2 protein will be central to understanding its role in initiating the formation of the ninefold symmetrical cartwheel of the procentriole. As no crystal structure of the full-length Ana2 protein has been determined, we turned to HDX-MS as a tool to study its conformational dynamics. HDX can assess the flexibility of protein regions by measuring the exchange of amide protons in the polypeptide chain with deuterium atoms after incubation with heavy water for predetermined times [40,41]. HDX-MS of Ana2 revealed that the protein is highly flexible. Even after a short time of incubation with D_2_O buffer (10 s), deuterium exchange was complete along almost the full length of the protein (figure 1*a*). Only a short region, amino acids (aa) 186–220, remained protected from exchange. This overlapped with a known C–C motif (aa195–229) [36] and was detected using an *in silico* C–C prediction algorithm (figure 1*a,b*). The high flexibility of Ana2 protein strongly suggests it is unstructured and belongs to the class of intrinsically disordered proteins (IDP) in accordance with an *in silico* IDP prediction algorithm (figure 1*c*).

**Figure 2.**
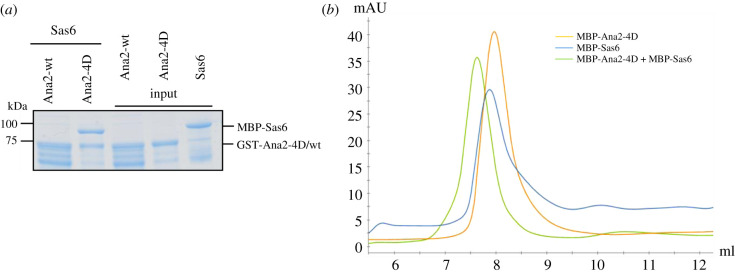
Ana2-4D interacts with Sas6 forming a stable complex *in vitro*. (*a*) SDS–PAGE of the binding assay in which Ana2-wt or Ana2-4D is the bait and Sas6 is the prey. Phosphomimetic version of Ana2 (Ana2-4D) binds Sas6 *in vitro*. (*b*) SEC of Ana2-4D, Sas6 and the Ana2-4D-Sas6 complex. Yellow, absorbance at 280 nm of MBP-Ana2-4D; blue, absorbance 280 nm of MBP-Sas6; green, absorbance 280 nm of MBP-Ana2-4D-MBP-Sas6 complex. Shift in retention time between single proteins and protein mixture indicates complex formation.

Recruitment of Sas6 to the procentriole is dependent upon the phosphorylation of four conserved serine residues in Ana2's STAN motif [14]. To be able to investigate this interaction by HDX, we set about reconstructing complex formation between Sas6 and a phosphomimetic variant of Ana2 (Ana2-4D) *in vitro*. To this end, we conducted an *in vitro* pull-down assay (binding assay) using baits of either wild-type (wt) GST-tagged Ana2 or GST-tagged Ana2 in which the STAN motif serines 318, 365, 370 and 373 were substituted by aspartic acid (Ana2-4D). Since Ana2-wt appeared to be less stable and degrades to a greater extent than Ana2-4D, we adjusted the amounts of full-length protein baits for the assay and both were used in comparable amounts. The experiment revealed that Ana2-4D could directly interact with Sas6, whereas Ana2-wt could not (figure 2*a*). We found that the complex formed between Ana2-4D and Sas6 was stable and could be fractionated by size exclusion chromatography (SEC) (figure 2*b*).

**Figure 3.**
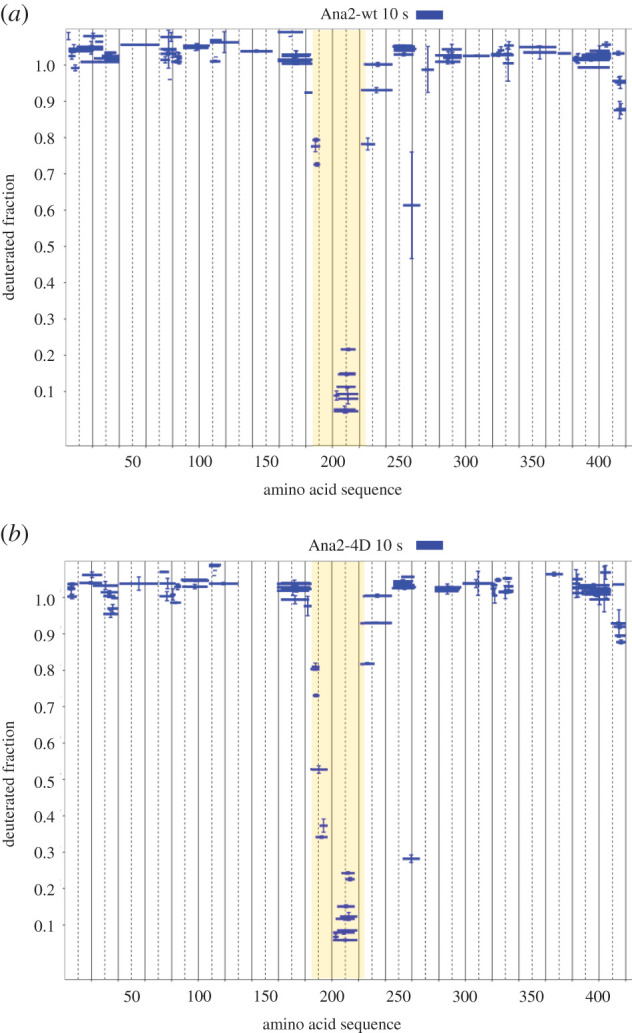
Structural comparison of Ana2-wt and Ana2-4D. (*a*) HDX pattern of Ana2-wt following a 10 s incubation with heavy water. Blue bars on Woods plots represent proteolytic peptides identified by mass spectrometry (MS) and positioned at the *x*-axis in relation to the Ana2 amino acid sequence, while their corresponding fraction of deuteration is shown at the *y*-axis (data taken from figure 1). (*b*) HDX pattern of Ana2-4D following a 10 s incubation with heavy water (mean of two experiments). Both HDX patterns (Ana2-wt and Ana2-4D) are consistently showing high flexibility along almost the full length of the protein, except for C–C region.

To determine whether the phosphomimetic form of Ana2 displayed any differences in structural stability from the wild-type form, we compared patterns of exchange in Ana2-wt with Ana2-4D by HDX-MS. This revealed that the HDX profiles of the two Ana2 variants after a 10 s incubation time with heavy water were almost identical (figure 3*a*,*b*). These experiments indicated similarly positioned short structured elements in both variants and similarly high flexibility of the remaining parts, even though their ability to bind Sas6 differs.

**Figure 4.**
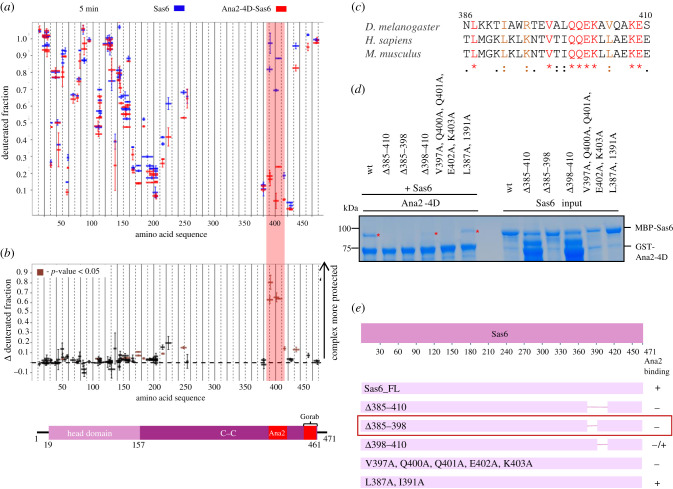
Sas6 interacts with Ana2-4D through its C-terminal region. (*a*) HDX pattern of Sas6 in complex with Ana2-4D, following a 5 min incubation with D_2_O. Shown are Sas6 peptides alone (blue bars) and when in complex with Ana2-4D (red bars). *x*-axis, position of peptides in amino acid sequence; *y*-axis, fraction of deuteration. The mean of two experiments is shown. Error bars show both values measured. (*b*) Upper panel: differences between deuteration of Sas6 peptides alone and in complex with Ana2-4D, derived by subtraction of deuteration levels shown in (*a*). Brown bars indicate peptides for which the differences measured in repeated experiments satisfied the Welsh *t*-test with *p* < 0.05. Pink highlighted box, peptides protected from exchange to the greatest extent when Sas6 is in complex with Ana2-4D (aa385–410). Lower panel: representation of Sas6 showing known domains: Head Domain, C–C motif, regions essential for the interaction with Ana2 and Gorab that were identified by HDX-MS. (*c*) Multiple sequence alignment (MSA) of Sas6 between amino acids 385 and 410. Residues in red, highest level of conservation between species and well conserved within an amino acid group. Residues in orange, high similarity and in the same aa group. MSA was performed using T-Coffee Expresso [45–49]. (*d*) SDS–PAGE of the binding assay in which Ana2-4D is the bait bound to Sas6 wild-type or its deletions and point mutants as prey. The binding assay shows the importance of Sas6 aa 385–410 for the interaction with Ana2-4D. (*e*) Schematic showing ability of Sas6 deletion and point mutants to bind Ana2 *in vitro*. Red box, the shortest region of Sas6 essential for Ana2 binding (aa385–398).

**Figure 5.**
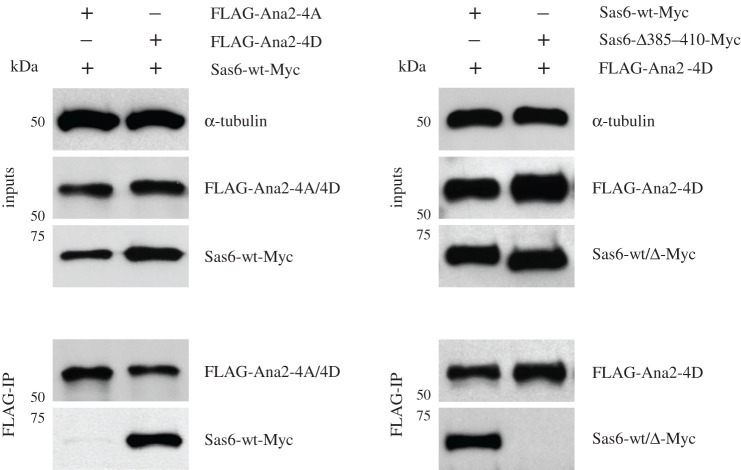
Sas6-Δ385–410 cannot interact with Ana2 *in vivo*. (*a*) The non-phosphorylatable version of Ana2 (FLAG-Ana2-4A) or the four aspartic acid substitution phosphomimetic mutant (FLAG-Ana2-4D) were transiently co-expressed with Myc-tagged wild-type Sas6 (Sas6-wt-Myc) in D.Mel-2 cells. Inputs and anti-FLAG immunoprecipitates were subjected to SDS–PAGE and western blotting to reveal the indicated antigens. (*b*) FLAG-Ana2-4D was co-expressed with either wild-type Myc-tagged Sas6 (Sas6-wt-Myc) or with the short-deletion Sas6 mutant version (Sas6-Δ385–410-Myc). Inputs and immunoprecipitates are displayed as in (*a*).

We next aimed to identify the region of Sas6 that is responsible for binding Ana2-4D. We therefore carried out HDX-MS to compare the deuterium exchange profile of Sas6 alone to Sas6 in complex with Ana2-4D. A comparison of the HDX profiles revealed reduced deuteration of Sas6 in the region aa 385–410, strongly suggesting the interaction surface encompasses this area (figure 4*a*,*b*). A multiple sequence alignment of this region comparing Sas6 homologues among several species revealed several highly conserved residues (figure 4*c*). To confirm the importance of this region of Sas6 for binding Ana2-4D, we designed a set of Sas6 deletions and point mutations in conserved amino acids for testing in an *in vitro* binding assay. This revealed that deletions aa385–410, aa385–398 and polyalanine mutant (V397A, Q400A, Q401A, E402A, K403A) abolish the interaction with Ana2-4D whereas deletion aa398–410 still binds Ana2-4D but weakly (figure 4*d*). Mutation of the two conserved residues L387A and I391A did not affect the ability of Sas6 to bind Ana2-4D (figure 4*d*). To validate these findings in a more physiological context, we co-transfected D.Mel-2 cells with FLAG-Ana2 (either the non-phosphorylatable 4A mutant or the 4D) and Sas6-Myc (either wt or Δ385–410). After FLAG-pulldowns, we confirmed that Sas6-wt could only interact with Ana2-4D but not with Ana2-4A (figure 5*a*). This verifies our previous findings that Ana2 and Sas6 can only associate following phosphorylation (in this case simulated by using the phosphomimetic version Ana2-4D) of Ana2's STAN motif by Plk4. Using this ‘activated’ Ana2-4D mutant protein, we then confirmed that it was only able to bind to Sas6-wt, but not to Sas6-Δ385–410 (figure 5*b*). Together, these results suggest that the entire region aa385–410 of Sas6 serves as an interaction surface for Ana2-4D.

**Figure 6.**
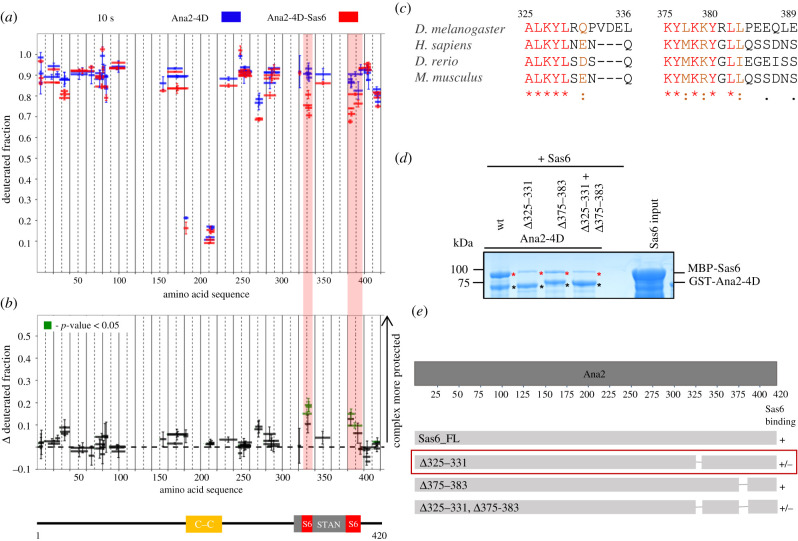
Ana2-4D interacts with Sas6 through its C-terminal region. (*a*) HDX pattern of Ana2-4D in complex with Sas6, following a 10 s incubation with D_2_O. Shown are Ana2-4D peptides alone (blue bars) and when in complex with Sas6 (red bars). *x*-axis, position of peptides in amino acid sequence; *y*-axis, fraction of deuteration. The mean of two experiments is shown. Error bars show both values measured. (*b*) Upper panel: differences between deuteration of Ana2-4D peptides alone and in complex with Sas6, derived by subtraction of deuteration levels shown in (*a*). Green bars indicate peptides for which the differences measured in repeated experiments satisfied the Welsh *t*-test with *p* < 0.05. Pink highlighted boxes, peptides protected from exchange to the greatest extent when Ana2-4D is in a complex with Sas6 (aa325–336 and 380–393). Lower panel: representation of Ana2 showing known domains: yellow box, structured region identified by HDX-MS covering C–C motif; grey box, STAN motif. (*c*) Multiple sequence alignment (MSA) of the Ana2 region between amino acids 325–336 and 375–389. Residues in red, the highest level of conservation between species and well conserved within an aa group. Residues in orange, high similarity and in the same aa group. MSA was performed using T-Coffee Expresso [45–49]. (*d*) SDS–PAGE of the binding assay in which Ana2-4D or Ana2-4D deletion mutants are the bait and Sas6 wild-type is the prey. (e) Schematic showing the ability of Ana2 deletion mutants to bind Sas6 *in vitro*. Red box, region of Ana2 important for Sas6 binding (aa325–331).

In the reciprocal analysis, we attempted to identify the region of the Ana2-4D HDX profile sensing the presence of Sas6 upon complex formation (figure 6). Upon 10 s incubation with D_2_O, we could observe mild, yet significant differences in deuterium uptake in two regions of Ana2-4D (aa325–336 and aa380–389) in a comparison of Ana2 alone or in complex with Sas6. To verify the importance of these two regions for Sas6 binding, we designed deletions within or flanking them based upon sequence conservation evident from multiple sequence alignment (figure 6*c*). Sas6 binding was strongly diminished by the aa325–331 deletion of Ana2-4D but less so by the aa375–383 deletion (figure 6*d*,*e*). A combination of these two deletions had a similar effect upon interaction with Sas6 as the single deletion aa325–331. Together, this indicates the importance of the 325–331 aa region but suggests additional sequences can mediate weak interactions.

**Figure 7.**
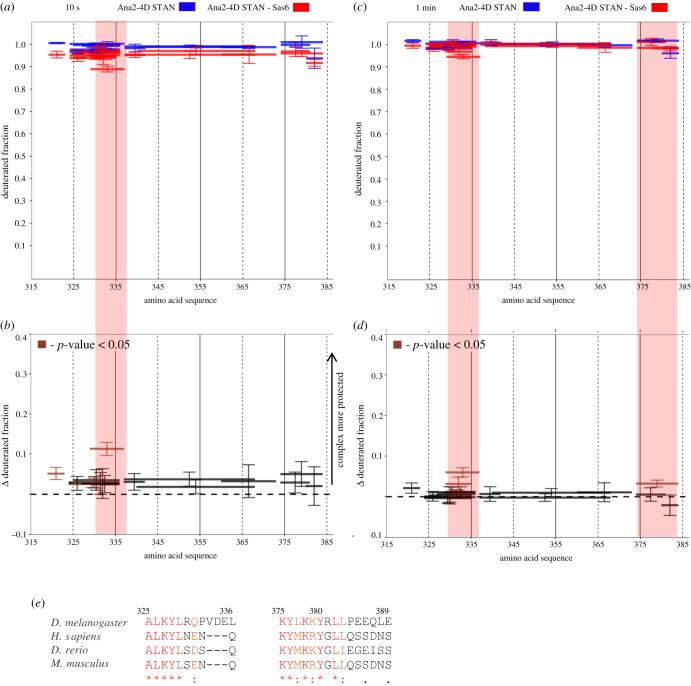
Ana2-4D STAN motif interacts with Sas6. (*a*) HDX pattern of Ana2-4D STAN motif in complex with Sas6 following a 10 s incubation with D_2_O. Ana2-4D STAN peptides alone (blue bars) and when in complex with Sas6 (red bars) are shown. *x*-axis, position of peptides in amino acid sequence; *y*-axis, fraction of deuteration. The mean of three experiments is shown. Error bars represent standard deviations. (*b*) Differences between deuteration of Ana2-4D STAN peptides alone and in complex with Sas6, derived by subtraction of deuteration levels shown in (*a*). Brown bars indicate peptides for which the differences measured in repeated experiments satisfied the Welsh *t*-test with *p* < 0.05. Pink highlighted box, peptides protected from exchange to the greatest extent when Ana2-4D STAN motif is in complex with Sas6 (aa329–336). (*c*) HDX pattern of Ana2-4D STAN alone (blue bars) and in complex with Sas6 (red bars), following 1 min incubation with D_2_O. The mean of three experiments is shown. (*d*) Differences between deuteration of Ana2-4D STAN peptides alone and in complex with Sas6, derived by subtraction of deuteration levels shown in (*c*). Pink highlighted box, peptides protected to the greatest extent when Ana2-4D STAN is in complex with Sas6 (aa329–336 and 374–384). (*e*) Multiple Sequence Alignment (MSA) of Ana2 region between amino acids 325–336 and 375–389. Residues in red, the highest level of conservation between species and well conserved within an aa group. Residues in orange, high similarity and in the same aa group MSA was performed using T-Coffee Expresso [45–49].

Since the phosphorylated STAN motif alone is able to bind Sas6, we carried out analogous experiments to follow HDX in a 69 aa Ana2-4D STAN motif fragment incubated alone or with Sas6 in D_2_O for 10 s or 1 min (figure 7). In accord with the HDX data obtained for full-length Ana2-4D with Sas6, this revealed slightly higher protection in the regions aa329–336 and aa374–384 when the Ana2-4D STAN motif was in complex with Sas6 (figure 7*a–e*). Thus, the mild stabilization of these two regions upon complex formation is reproducible not only with Ana2-4D-FL, but also with the Ana2-STAN-4D motif alone, wherein these two stabilized regions reside. Interestingly, these regions (aa325–336 and aa380–389) seem to be the most highly conserved portions of the STAN motif with each lying adjacent to a stretch of serine residues (S318 and S365;S370;S373, respectively), which are phosphorylated by Plk4 to trigger Ana2's interaction with Sas6. Taken together, this leads us to hypothesize that even though the entire STAN motif, and the phosphorylation of four conserved serines within it, seems to be important for the Ana2–Sas6 interaction, the two regions identified by HDX have a more direct involvement in the physical interaction between Ana2 and Sas6.

**Figure 8.**
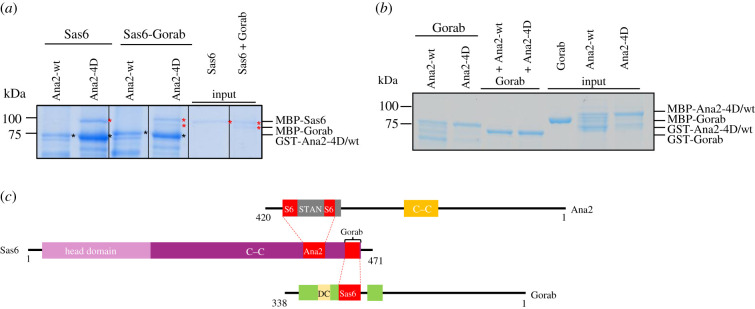
Sas6 can bind both Ana2 and Gorab simultaneously. (*a*) SDS–PAGE of the binding assay in which Ana2-wt or Ana2-4D is bait and Sas6 or Sas6–Gorab complex is the prey. Sas6 can bind Gorab and Ana2-4D simultaneously *in vitro*. (*b*) SDS–PAGE of the binding assay using either Ana2-wt or Ana2-4D as bait and Gorab as prey and *vice versa* showing Ana2 cannot directly interact with Gorab. (*c*) Representations of Ana2, Sas6 and Gorab showing known protein domains; Ana2: yellow box, structured region identified by HDX-MS covering C–C motif; grey box, STAN motif; Sas6: pink box, Head Domain; purple box, C–C motif; Gorab: green boxes, structured region identified by HDX-MS, yellow box, dimerization core. Red boxes and dashed lines indicate regions essential for the interactions between the three proteins identified by HDX-MS.

The Sas6 dimer forms a single unit in the ninefold radially symmetric structure that constitutes the procentriole's cartwheel. The two N-terminal globular parts of the dimer interact with adjacent subunits in the hub of this structure with the interacting C–C forming the spokes. Our current findings identify the segment on this spoke to which Ana2 binds and which is required to initiate procentriole formation. We recently discovered that the protein Gorab, itself essential for centriole duplication, also interacts with the Sas6 C–C region [27]. This led us to ask whether Sas6 is able to interact with both proteins at the same time or whether they show competition for binding. To address this, we performed a binding assay by immobilizing either Ana2-wt or Ana2-4D and determining whether Sas6 alone or Sas6–Gorab complex could be captured. This experiment revealed that Sas6 is able to bind both proteins at the same time indicating that Ana2 and Gorab do not compete for Sas6 binding (figure 8*a*). Finally, we tested if Ana2 could bind Gorab. A binding assay using either Ana2-wt or Ana2-4D as bait and Gorab as prey and *vice versa* showed that Ana2 could not directly interact with Gorab (figure 8*b*). Our finding that Sas6 can bind both Ana2 and Gorab simultaneously is in line with our recent mapping of the interface between Sas6 and Gorab within 20 residues (amino acids 440–460) towards the C-terminus of Sas6.

## 3. Material and methods

### 3.1. Plasmids

All expression vectors were generated using the Gateway system (Invitrogen). For MBP and GST tagging, we used pKM596 (Addgene) and pDEST15 (Invitrogen), respectively, as destination vectors. Flag and Myc tagging of Ana2 and Sas6 proteins for the co-immunoprecipitation experiments was achieved using the pAFW, and pAWM destination vectors from the Drosophila Gateway Vector Collection (https://emb.carnegiescience.edu/labs/murphy/Gateway%20vectors.html). The QuickChange Mutagenesis Kit (Agilent) was used to introduce all deletions and amino acid substitution mutations. The constructs were verified by DNA sequencing.

### 3.2. Protein expression and purification

Recombinant proteins were expressed in *Escherichia coli* strain *Rosetta(DE3*) (Thermo Fisher) following standard procedures. Briefly, bacteria were transformed with recombinant plasmids encoding the desired proteins and cultured at 37°C to *A*_600_ of approximately 0.5–0.7 in Terrific Broth supplemented with appropriate antibiotics. Protein expression was induced with 0.5 mM isopropyl-b-d-1-thiogalactopyrano-side (IPTG) at 20°C overnight. Bacterial cells were harvested, resuspended in buffer A (20 mM Tris–HCl pH 7.5, 150 mM NaCl, 5% (v/v) glycerol, 1 mM dithiothreitol (DTT)) supplemented with EDTA-free protease inhibitor cocktail (Roche) and incubated on ice for 30 min. Cells were lysed by sonication and clarified by centrifugation at 15 000*g* for 15 min at 4°C. The cleared lysates were incubated with amylose resin (NEB) or glutathione supharose 4B resin (GE healthcare), for MBP- or GST-tagged proteins respectively, for 2 h at 4°C. Beads with bound proteins were washed three times for 10 min with 30 resin volumes of buffer A. Bound proteins were eluted with buffer A supplemented with 20 mM maltose or 10 mM glutathione.

### 3.3. *In vitro* complex formation

Proteins were expressed in *E. coli*, and purified as described in the ‘Protein expression and purification’ section. MBP-Ana2-4D was then mixed with MBP-Sas6, incubated for at least 1 h on ice and then loaded on a Superdex 200 10/300 (GE Healthcare) column. SEC was run and fractions were collected and analysed by SDS–PAGE and PageBlue protein staining (Thermo Fisher).

### 3.4. Size exclusion chromatography

For SEC, we used Superose6 10/300 (GE Healthcare) or Superdex 200 10/300 (GE Healthcare) columns pre-equilibrated with buffer (20 mM Tris–HCl pH 7.5, 150 mM NaCl, 1 mM dithiothreitol (DTT)). Affinity-purified protein samples were loaded onto the columns and SEC was run at a 0.5 ml min^−1^ flow rate at 4°C. The elution of proteins was monitored at 280 nm. Fractions were collected and analysed by SDS–PAGE and PageBlue protein staining (Thermo Fisher). For HDX-MS studies, the principal fractions having the highest protein concentration were used.

### 3.5. Hydrogen–deuterium exchange mass spectrometry

Peptide lists were established by diluting 5 µl of each analysed protein 10-fold into a non-deuterated buffer (20 mM Tris–HCl pH 7.5, 150 mM NaCl, 1 mM DTT). The sample (50 µl) was acidified by mixing with 10 µl of ‘Stop’ buffer (2 M glycine pH 2.5, 1.5 M Urea, 250 mM TCEP or 2 M glycine pH 2.5 for Ana2-4D STAN) and digested offline in the ThermoMixer (Eppendorf) for 30 s at 1°C with 2 µl of protease (*Aspergillus* saitoi, type XIII (Sigma)) and then injected into a nanoACQUITY UPLC system (Waters) equipped with an HDX Manager system (Waters) with the column outlet coupled directly with a SYNAPT G2 HDMS mass spectrometer, followed by online digestion using an immobilized pepsin column (Porozyme, ABI) with 0.07% formic acid in water as the mobile phase (flow rate 200 µl min^−1^). Digested peptides were trapped on a C18 column (UPLC BEH C18 Van-Guard Pre-column 1.7 µm, 2.1 × 5 mm, Waters) and then directed into a reverse phase column (UPLC BEH C18 column 1.7 µm 2.1 × 100 mm, Waters) with a 10–35% gradient of acetonitrile in 0.1% formic acid at 90 µl min^−1^ using nanoACQUITY Binary Solvent Manager. The total time for a single run was 12 min. All capillaries, valves and columns were maintained at 0.5°C inside an HDX cooling chamber, while the pepsin column was kept at 20°C inside the temperature controlled digestion compartment. Leucine–enkephalin solution (Sigma) was used as a Lock mass. For protein identification, mass spectra were acquired in MSE mode over the *m/z* range of 50–1950. The spectrometer parameters were as follows: ESI positive mode, capillary voltage 3 kV, sampling cone voltage 35 V, extraction cone voltage 3 V, source temperature 80°C, desolvation temperature 175°C and desolvation gas flow 800 l/ h. Peptides were identified using Protein Lynx Global Server (PLGS) software (Waters). The list of identified peptides containing peptide *m/z*, charge and retention time was further processed with the Dynamx v. 3.0 program (Waters).

For HDX experiments, protein samples were diluted in the Reaction buffer containing 99.8% D_2_O (Cambridge Isotope Laboratories). Five microlitres of protein stock solution was mixed with 45 μl D_2_O Reaction buffer and an exchange reaction was carried out for a specific time period (either 10 s, 1 or 5 min) on ice. The exchange was quenched by reducing the pH to 2.5 by adding the reaction mixture into an Eppendorf tube containing ice-cold Stop buffer (2 M glycine pH 2.5, 1.5 M Urea, 250 mM TCEP or 2 M glycine pH 2.5 for Ana2-4D STAN). Immediately after quenching, samples were snap-frozen in liquid nitrogen and stored at −80°C until analysed. Quenched samples were rapidly thawed, digested offline as described above and manually injected into the nanoACQUITY UPLC system. Further digestion, LC and MS analysis were carried out exactly as described for the non-deuterated sample. For the out-exchange control experiment, measuring the maximum exchange for a given peptide, the 5 µl protein stock was mixed with 45 µl of D_2_O Reaction buffer, incubated for 24 h at RT, mixed with Stop buffer and analysed as described above. The deuteration level in the out-exchange control experiment was calculated and denoted as 100% exchange (*M*_ex__100_). HDX experiments were repeated at least three times. Experiments were repeated using either different overexpression batches (biological replicates) or the same batch (technical replicates).

### 3.6. HDX-MS data analysis

A peptide list was created for each protein using the DynamX 3.0 software based on PLGS peptide identifications, with following acceptance criteria: minimum intensity threshold, 1000–3000; minimum fragmentation products per amino acids for precursor, 0.3 or 0.25; maximum mass difference between measured and theoretical value for parent ions, 10 ppm. Analysis of the isotopic envelopes in DynamX 3.0 software was carried out using the following parameters: retention time deviation ± 18 s; *m/z* deviation ± 15 ppm; drift time deviation ±2 time bins. Centroids of the mass envelopes were obtained. The values reflecting the experimental mass of each peptide in all possible states, replicates, time points and charge states were exported from the DynamX 3.0 and further data analysis was carried out using in house scripts written in R (http://www.R-project.org). The deuterated fraction (*D*) was calculated with the following formula: *D* = (*M*_ex_ – *M*_ex0_) ÷ (*M*_ex100_ – *M*_ex0_), where *M*_ex0_ indicates the average peptides mass with 0% exchange and *M*_ex100_ indicates the average peptide mass measured in out-exchange control, respectively. Error bars for fraction exchanged represent standard deviations calculated from independent replicates. The difference in the fraction exchanged (Δ deuterated fraction) was calculated by subtracting the fraction-exchanged values for peptides in the selected state from the values for the same peptides in the control state. The error bars were calculated as the square root of the sum of the variances from compared states. Student's *t*-test for independent measurements with unequal variances and unequal sample sizes (also known as Welsh *t*-test) was carried out to evaluate differences in fraction exchanged between the same peptides in two different states.

### 3.7. *In vitro* pull-down assay (binding assay)

*In vitro* pull-down assays were carried out by incubating the lysate containing bait protein on Glutathione-Sepharose 4B (GE Healthcare) resin. After mixing by rotation for 1 h at 4°C, the beads were washed three times for 10 min with buffer B containing 20 mM Tris–HCl pH 7.5, 250 mM NaCl, 5% (v/v) glycerol, 1 mM dithiothreitol (DTT), 0.5% (v/v) Triton. Next, the lysate with prey MBP-tagged protein was added and incubated for 1 h at 4°C, followed by 3 × 10 min washes with buffer B. The proteins were eluted by boiling in Laemmli sample buffer and analysed by SDS–PAGE with PageBlue protein staining (Thermo Fisher).

### 3.8. Co-immunoprecipitation

Co-immunoprecipitation was performed as previously described [14]. In brief, D.Mel-2 cells were co-transfected with constitutively driven constructs encoding 3xFLAG-Ana2 (4A or 4D) and Sas6-6xMyc (wt or Δ385-410) for 24 h. Cells were treated with 25 µM MG132 (Sigma) for 5 h, collected and lysed in 1 ml ice-cold buffer containing 50 Na-HEPES pH 7.5, 150 mM NaCl, 2 mM MgCl2,0.5 mM Na-EGTA pH 8.0, 1 mM DTT, 0.1% NP-40, 5% glycerol, 1 mM PMSF and EDTA-free complete protease inhibitor cocktail (Roche) by passing 6× through a prechilled G25 needle. The lysates were clarified by centrifugation (2°C, 12 000*g*, 10 min) and the supernatants were mixed with anti-FLAGM2 magnetic beads (Sigma) for 2–4 h at 4°C. After washing four times, the proteins were eluted from the beads by Laemmli sample buffer and subjected to SDS–PAGE followed by immunoblotting.

## Data accessibility

The raw data are available from the authors.
